# A novel Cherenkov radiation removal method for plastic scintillator detectors in a 0.35 T MR‐Linac

**DOI:** 10.1002/acm2.70202

**Published:** 2025-08-15

**Authors:** Mateb Al Khalifa, Tianjun Ma, Haya Aljuaid, Siyong Kim, William Y. Song

**Affiliations:** ^1^ Department of Radiation Oncology Virginia Commonwealth University Richmond Virginia USA

**Keywords:** Cherenkov radiation, MR‐Linac, plastic scintillation detectors, QA, small field dosimetry

## Abstract

**Purpose:**

This study evaluates methods for removing Cherenkov radiation (CR) from plastic scintillation detectors (PSDs), focusing on constraints specific to a 0.35 T MR‐Linac system.

**Methods:**

Five CR‐removal methods were examined: cross calibration, fiber alone, multiloop, collimator rotation, and couch rotation. The first three (cross calibration, fiber alone, and multiloop) were tested on a 0.35 T MR‐Linac (ViewRay Inc., USA) using the BluePhysics PSD (Blue Physics LLC, USA). These methods do not require collimator or couch rotation. The remaining two methods (collimator rotation and couch rotation) were tested on a Varian TrueBeam (Varian Medical Systems, USA) for comparison. Measurements were performed under various setup configurations, and Cherenkov radiation extraction (CRE) values were calculated to determine each method's effectiveness.

**Results:**

The multiloop approach yielded a CRE of 0.7288, making it the most practical and robust for MR‐Linac constraints because it requires neither collimator nor couch rotation. The cross calibration and fiber alone methods produced CRE values of 0.7318 and 0.7569, respectively. Collimator rotation gave 0.7255, comparable to multiloop. In contrast, couch rotation resulted in 0.7489 but exhibited more variability, suggesting lower reliability.

**Conclusion:**

The multiloop method emerged as the most practical and robust technique for CR removal in 0.35 T MR‐Linac systems. Its simplicity and compatibility with MR‐Linac design constraints make it a highly effective approach for CR removal in PSD‐based radiotherapy applications.

## 1 INTRODUCTION

The use of plastic scintillation detectors (PSDs) in magnetic resonance guided radiotherapy (MRgRT) systems marks a significant advancement in radiotherapy. Since plastic scintillators were first introduced for high‐energy radiotherapy applications, considerable attention has been paid to their properties.^1^ These include water equivalence (eliminating the need for heterogeneity corrections), a linear relationship between emitted light and detected charge energy, rapid signal decay, and high transparency.^2^, ^3^, ^4^, ^5^ In addition, their small volume reduces partial volume effects, which is especially beneficial in small‐field dosimetry.^6^, ^7^, ^8^, ^9^, ^10^

When a PSD is irradiated, charged particles can travel faster than the local speed of light in both the optical fiber and the plastic scintillator fiber, generating Cherenkov radiation (CR).^11^, ^12^, ^13^ Although CR can form the basis of certain radiation measurements,^14^, ^15^, ^16^, ^17^, ^18^ it acts as noise when the objective is to measure the scintillator signal. The CR signal overlaps with the PSD signal, affecting measurement accuracy; it is therefore considered a contaminant generated by both the sensitive volume of the PSD and the optical fiber, requiring removal.

Compared to other radiation detectors, PSDs are preferable in MR‐Linac systems because the presence of a magnetic field impacts detector performance.^19^, ^20^, ^21^, ^22^ Notably, in ionization chambers, the Lorentz force deflects secondary electrons, which can significantly affect dose readings, especially given the density difference between the air in the chamber cavity and the chamber walls.^23^, ^24^ The magnet also affects other detectors, including diode and diamond detectors.^25^ In contrast, when a detector is water‐equivalent, the magnet's impact on dose response is reduced because equilibrium is reached between the detector and its surroundings.^25^, ^26^, ^27^

This study focuses on the application of PSDs in a 0.35 T MR‐Linac system (ViewRay Inc., USA). We evaluated several CR‐removal techniques using a 1 mm BluePhysics Plastic Scintillator Detector (BP‐PSD) (Blue Physics LLC, USA). The detector is classified as a dose‐based, high‐temporal‐and‐spatial resolution PSD that enables measurement of every single Linac pulse with ± 700 µs temporal accuracy. It consists of two identical optical fibers bonded together: one has a plastic scintillator at its distal end, and the other does not. The plastic scintillator fiber is cylindrical (1 mm length, 1 mm diameter, 0.785 mm^3^ volume) and made of polystyrene. Both optical fibers are 0.25 mm in diameter and 20 m in length, composed of polymethylmethacrylate (PMMA). The fiber with the scintillator carries both the CR signal and the desired scintillation signal (chPSD), while the fiber without the scintillator measures only the CR signal (chCR). Because CR is produced almost equally in both fibers, the signal from the non‐scintillating fiber is used to estimate and subtract the CR component from the scintillator fiber.

The BP‐PSD has been investigated in both MR‐Linac and conventional Linac systems. Ferrer et al. studied the BP‐PSD in a 1.5 T MR‐Linac,^28^ examining its repeatability, dose‐response linearity, dose‐rate dependence, angular dependence, temperature dependence, field output factor (FOF), and suitability for percentage depth dose (PDD) and profile measurements. They observed reliable performance in MR‐Linac environments, including stable repeatability and linearity with no sensitivity to dose rate or temperature. Similarly, Das et al. characterized the BP‐PSD in a conventional Linac, evaluating dose linearity, dose‐rate dependence, angular dependence, temperature dependence, PDD, beam profiles, and FOF, and found it suitable for routine measurements.^29^

Several studies have explored different CR‐removal methods, each with distinct advantages and drawbacks.^28^, ^29^, ^30^, ^31^, ^32^, ^33^, ^34^, ^35^ Given the unique design of the 0.35 T MR‐Linac, this study examines three CR‐removal methods that do not require rotating either the collimator or the couch. The first is cross calibration, a conventional approach.^28^, ^29^ The second irradiates only the optical fiber, leaving the plastic scintillator outside the field. In addition, we propose a multiloop method, in which both the scintillator fiber and the optical fiber are irradiated by looping the fiber multiple times over repeated irradiations. For comparison, we also evaluated CR signal removal on a Varian TrueBeam (Varian Medical Systems, USA) by rotating only the collimator or only the couch. Our aim is to assess each method's advantages, limitations, and efficiency in removing CR signals.

## 2 METHODS AND MATERIALS

The BP‐PSD manufacturer refers to the process of removing CR as the adjacent channel ratio (ACR). However, to generalize this concept, we use the term Cherenkov radiation extraction (CRE) in this study.

### 2.1 Cross calibration method

Measurements were conducted using a PTW BeamScan MR water tank (PTW Dosimetry, Germany) with the 0.35 T MR‐Linac system. The water tank was placed on the treatment couch at the virtual isocenter, located outside the 0.35 T MR‐Linac bore at a distance of 155 cm from the treatment isocenter. The BP‐PSD was mounted in the detector holder and positioned at the water surface level (via visual inspection) so that its midpoint lay exactly halfway between being inside and outside the water, in accordance with AAPM TG‐106.^36^ External lasers intersecting at the virtual isocenter were used to align the detector along the inline and crossline axes.

Next, the BP‐PSD was moved to a depth of 5 cm [source to surface distance (SSD)  =  85 cm, source to axis distance (SAD)  =  80 cm], which corresponds to the virtual isocenter. The water tank was then moved from the virtual isocenter into the 0.35 T MR‐Linac bore, placing the detector at the treatment isocenter. To ensure precise detector positioning in the radiation field, a 0.83 cm × 0.83 cm field was scanned to identify the beam center. Once found, the detector was ready for measurement.

For the cross calibration, three 100 MU readings were taken for two field sizes (9.96 cm × 9.96 cm and 3.32 cm × 3.32 cm). Since the BP‐PSD has two optical fibers—one that detects both scintillation and Cherenkov (PSD channel) and one that detects Cherenkov only (CR channel)—the Cherenkov radiation extraction (CRE) was calculated as

(1) $$\mathrm{CRE}=\frac{R_{chPSD,9.96\;\times \;9.96}\;\times \;FOF-\;R_{chPSD,3.32\;\times \;3.32}}{R_{chCR,9.96\;\times \;9.96\;}\times \;FOF-\;R_{chCR,3.32\;\times \;3.32}}$$

where R_chPSD, 9.96 × 9.96_ and R_chPSD, 3.32 × 3.32_ denote the PSD‐channel readings for the larger and smaller fields, respectively, and R_chCR, 9.96 × 9.96_ and R_chCR, 3.32 × 3.32_ are the corresponding CR‐channel readings. FOF denotes the FOF, and the values for the 9.96 cm × 9.96 cm and 3.32 cm × 3.32 cm fields were obtained from the 0.35 T MR‐Linac Monte Carlo–based treatment planning system.

### 2.2 Fiber alone method

Measurements for the fiber‐alone method were performed on the 0.35 T MR‐Linac using the same PTW BeamScan MR water tank setup described in Section 2.1 to ensure detector alignment at the virtual isocenter. A depth of 5 cm (SSD  =  85 cm, SAD  =  90 cm) was used, and the tank was then moved from the virtual isocenter to the treatment isocenter.

To isolate the optical fiber, a 4.15 cm × 4.15 cm field was employed. Along the inline axis, the detector was moved from the field center (0 cm) in 1 cm increments until the plastic scintillator reading approached zero at −6 cm, confirming both the plastic scintillator tip and optical fibers were outside the field. The detector was then shifted to +6 cm so only the optical fibers remained inside the field. For the lateral axis, 0 cm was defined as the radiation field center. The detector was then shifted ± 2 cm in 0.1 cm increments to keep the optical fibers within the radiation field while the scintillator tip remained outside.

Once fiber‐only positioning was verified, three 100 MU readings were taken, and their average was used to calculate the CRE

(2) $$\mathrm{CRE}=\frac{R_{chPSD,4.15\;\times \;4.15}\;}{R_{chCR,4.15\;\times \;4.15\;}}$$

where R_ch,PSD, 4.15 × 4.15_ is the reading from the optical fiber connected to the plastic scintillator and R_chCR, 4.15 × 4.15_ is the reading from the optical fiber without the scintillator tip.

### 2.3 Multi‐loop method

The BP‐PSD was positioned in the PTW BeamScan MR water tank at the 0.35 T MR‐Linac virtual isocenter, using the same alignment approach as in Section 2.1. A depth of 5 cm (SSD  =  85 cm, SAD  =  90 cm) was set, and the tank was then moved from the virtual isocenter to the treatment isocenter. Scanning a 0.83 cm × 0.83 cm field at 5 cm depth ensured accurate detector placement along the inline and crossline axes.

**FIGURE 1:**
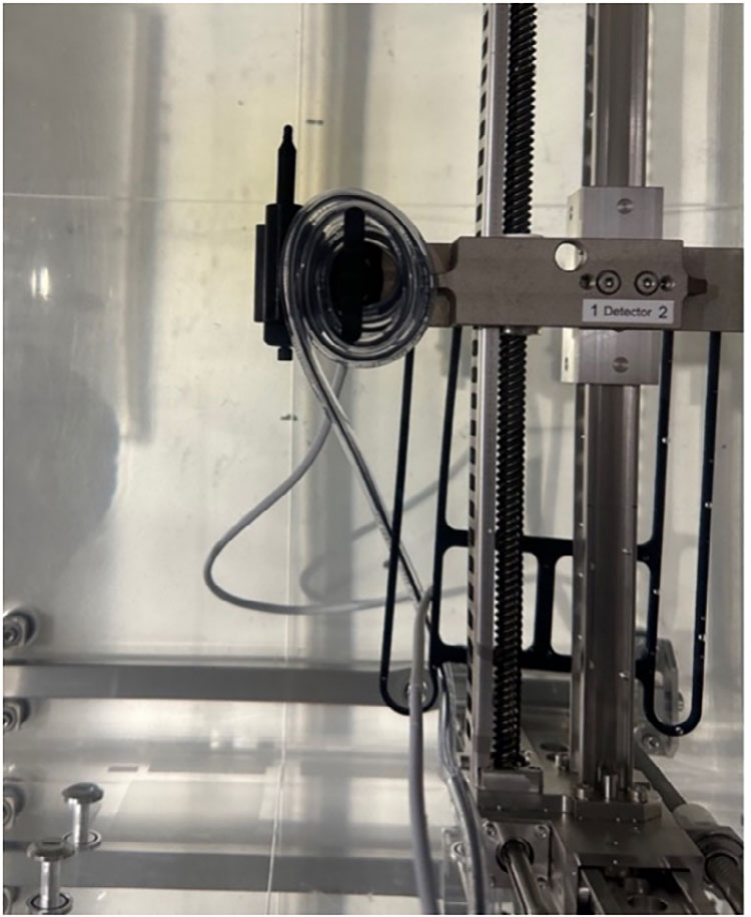
Illustration of how the optical fiber was looped alongside the PSD from loop 1 to loop 8. The optical fiber was wound a specified number of times for each loop configuration.

For the multiloop method, an initial measurement (loop 0) was taken at 100 MU with a 9.96 cm × 9.96 cm field and no loops in the optical fiber. The fiber was then looped once (loop 1) and reirradiated under the same conditions. This process was repeated for loops 2 through 8, with three readings acquired and averaged at each loop count. Figure 1 illustrates how the optical fiber was arranged for looping. To obtain the CRE, the chCR signals were plotted on the x‐axis against the chPSD signals on the y‐axis for all loops. The slope of the resultant linear relationship provided the CRE value.

### 2.4 Collimator and couch rotation methods

These two methods were carried out on a TrueBeam linear accelerator (Varian, USA). A 5 cm slab of solid water was placed at the center of the crosshair on the treatment couch to provide sufficient backscatter. The BP‐PSD was then positioned at the crosshair center on the solid water at isocenter. Along the crossline direction, the field size was set to 3 cm, with the BP‐PSD lying in the middle (1.5 cm from each side). Along the inline direction, the field size was set to 6 cm so that the tip of the BP‐PSD was 1.5 cm toward the gantry and 4.5 cm away from it. This setup created a 3 cm × 6 cm rectangular field, with the BP‐PSD at one edge. A 1.5 cm bolus was placed on top of the BP‐PSD so that the detector was at a 1.5 cm depth (d_max_ for a 6 MV FFF beam). The SSD was 98.5 cm and the SAD was 100 cm. Two different approaches, collimator rotation and couch rotation, were performed under these conditions.

#### 2.4.1 Collimator rotation

The collimator was rotated from 0° to 90° in 10° increments, while the gantry and couch remained at 0°. At a collimator angle of 0°, 100 MU were delivered. Afterward, the collimator was rotated by 10°, and another 100 MU were delivered. This continued until 90°, with readings recorded at each increment.

**FIGURE 2:**
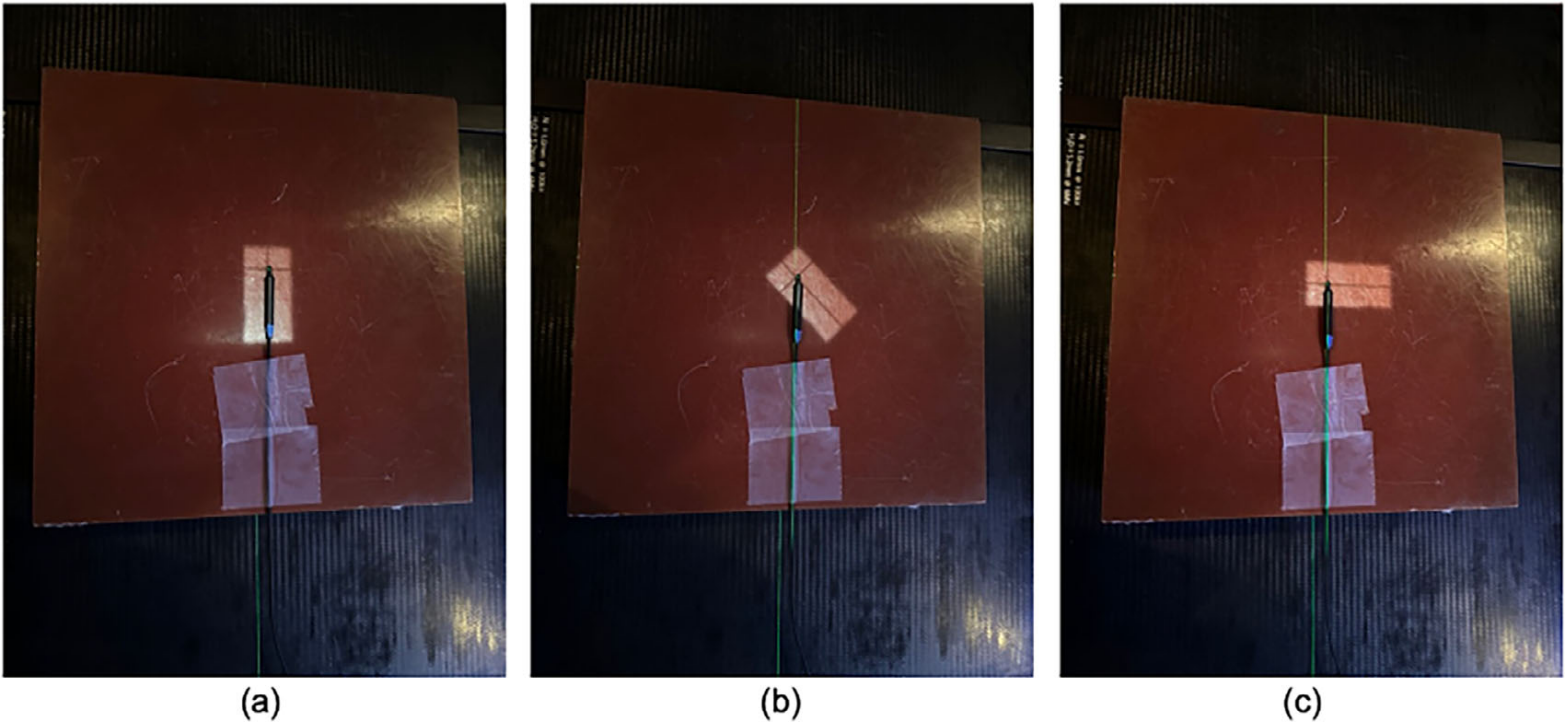
Field size orientation at different collimator angles while the couch remains fixed. Panels (a), (b), and (c) correspond to collimator angles of 0°, 45°, and 90°, respectively. Measurements were taken from 0° to 90° in 10° increments; panel (b) is shown only as an illustrative example, and no measurement was specifically acquired at that angle.

Figure 2 illustrates the field's orientation at different collimator angles, while keeping the detector's position fixed on the rotation axis. To determine the CRE, a graph was plotted with chCR on the x‐axis and chPSD on the y‐axis; the slope of this plot gives the CRE value.

#### 2.4.2 Couch rotation

**FIGURE 3:**
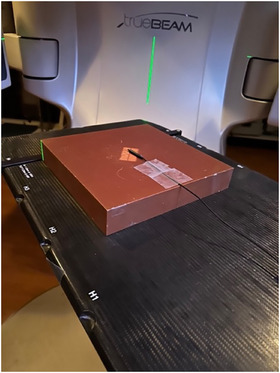
Illustration of the couch rotation method. The field size is shown at a collimator angle of 0°, while the couch is rotated from 0° to 90° in 10° increments for each measurement.

For the couch rotation approach, the collimator and gantry angles remained fixed at 0°, and only the couch was rotated from 0° to 90° in 10° increments. Figure 3 shows the couch rotation setup while holding the collimator angle constant. At each couch angle, measurements were taken and recorded. To find the CRE, a graph was plotted with chCR on the x‐axis and chPSD on the y‐axis. The slope of this plot provides the CRE value.

## 3 RESULTS

### 3.1 Cross calibration method

Table 1 presents the average values (based on three readings) for the number of pulses, the detector charge, and the Cherenkov charge for two field sizes (3.32 cm × 3.32 cm and 9.96 cm × 9.96 cm). These averages are shown with their corresponding standard deviations for each measured field size. From the TPS, the FOF was 0.911. Using Equation (1), the resulting CRE from this method was 0.7318.

**TABLE 1:** Cross‐calibration method results for two field sizes.

Field size (cm^2^)	Avg number of pulses	Ave detector charge (nC)	Ave Cherenkov charge (nC)	FOF (TPS)	CRE
**3.32 × 3.32**	1212.00 ± 0.00	96.51 ± 0.44	17.78 ± 0.34	0.911	0.7318
**9.96 × 9.96**	1210.33 ± 1.15	128.04 ± 0.15	49.71 ± 0.15		

The average (Avg) number of pulses, average detector charge, and average Cherenkov charge are listed with their respective standard deviations. The FOF values are taken from the 0.35 T MR‐Linac Monte Carlo–TPS. The CRE was computed using Equation (1).

### 3.2 Fiber alone method

Table 2 shows the values measured by the fiber‐alone method, including the average number of pulses, detector charge, and Cherenkov charge, along with their standard deviations. The resulting CRE, calculated via Equation (2), was 0.7569.

**TABLE 2:** Results of the fiber‐alone method. The average (Avg) number of pulses, average detector charge, and average Cherenkov charge are listed with their respective standard deviations.

Field size (cm^2^)	Avg number of pulses	Avg detector charge (nC)	Avg Cherenkov charge (nC)	CRE
**4.15 × 4.15**	1210.67 ± 1.53	39.04 ± 0.12	51.58 ± 0.31	0.7569

The CRE was calculated according to Equation (2).

### 3.3 Multiloop method

**FIGURE 4:**
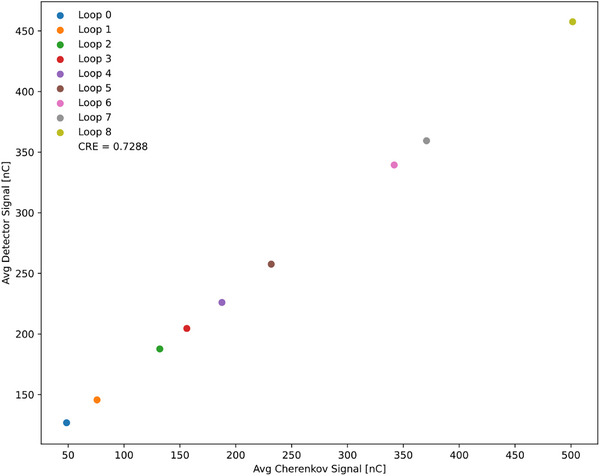
Relationship between the average Cherenkov signals on the x‐axis and the average detector signals on the y‐axis in the multiloop method. The CRE value was determined from the slope of the resulting linear plot.

Results from the multiloop method are listed in Table 3, indicating how many loops were applied in each measurement (0 indicates no loop, and 1 through 8 indicates the loop count). Each entry includes the average number of pulses, detector charge, Cherenkov charge, and their standard deviations. The CRE was determined from the plot in Figure 4, where the x‐axis represents the average Cherenkov charge (chCR) and the y‐axis represents the average detector charge (chPSD). The slope of the linear relationship was 0.7288.

**TABLE 3:** Values obtained using the multiloop method, showing the loop number and the corresponding average (Avg) number of pulses, detector charge, and Cherenkov charge, each listed with their respective standard deviations.

Number of loop	Avg number of pulses	Avg detector charge (nC)	Avg Cherenkov charge (nC)	CRE
**0**	1213.00 ± 1.00	126.81 ± 0.18	48.56 ± 0.05	0.7288
**1**	1213.67 ± 1.53	145.66 ± 0.22	75.81 ± 0.10	
**2**	1212.33 ± 0.58	187.68 ± 0.25	132.00 ± 0.28	
**3**	1215.00 ± 0.00	204.57 ± 0.79	156.17 ± 0.94	
**4**	1217.00 ± 0.00	226.04 ± 0.39	187.64 ± 0.18	
**5**	1212.00 ± 1.00	257.62 ± 0.40	231.74 ± 0.16	
**6**	1216.33 ± 1.53	339.45 ± 0.64	341.80 ± 0.52	
**7**	1211.67 ± 1.15	359.42 ± 0.68	370.75 ± 0.74	
**8**	1219.33 ± 2.08	457.56 ± 0.89	501.52 ± 0.85	

Loop 0 indicates that no loop was applied, while loops 1 through 8 specify the number of loops used. The CRE was determined from Figure 4. All measurements were performed with a 9.96 cm × 9.96 cm field size.

### 3.4 Collimator rotation method

**FIGURE 5:**
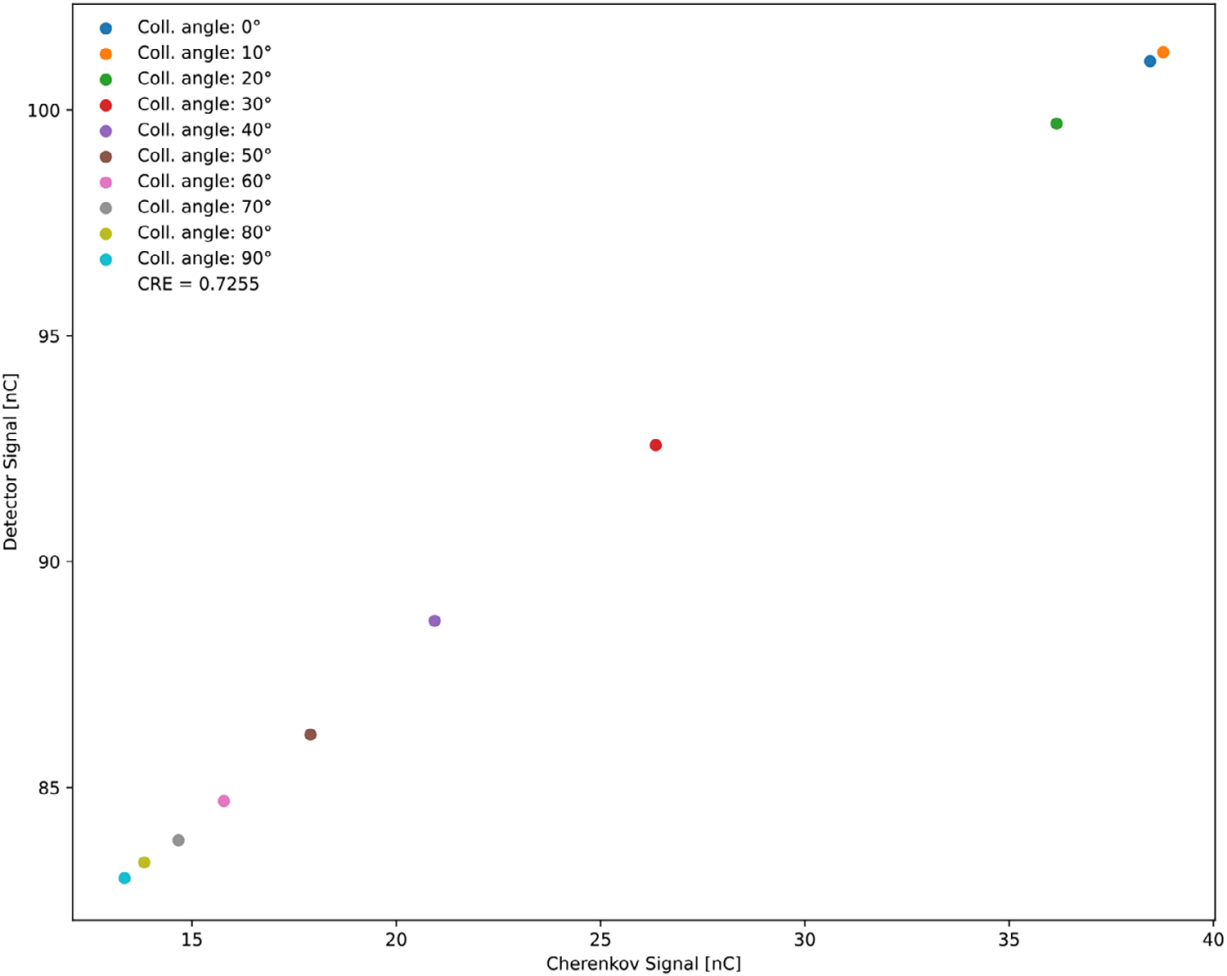
Relationship between Cherenkov signals (x‐axis) and detector signals (y‐axis) for each collimator angle in the collimator‐rotation method performed on the Varian TrueBeam. The slope of the linear plot represents the CRE.

The results of the collimator rotation method are shown in Table 4, covering angles from 0° to 90° in 10° increments. Each row displays the number of pulses, detector charge, and Cherenkov charge measured once per angle. The CRE, found to be 0.7255, was calculated from Figure 5 by plotting Cherenkov charges on the x‐axis against detector charges on the y‐axis.

**TABLE 4:** Measurement values from the collimator‐rotation method, including the collimator angle, number of pulses, detector charge, and Cherenkov charge.

Collimator angle	Numbers of pulses	Detector charge (nC)	Cherenkov charge (nC)	CRE
**0**	1326	101.08	38.45	0.7255
**10**	1324	101.28	38.77	
**20**	1324	99.70	36.16	
**30**	1325	92.58	26.35	
**40**	1327	88.69	20.94	
**50**	1325	86.17	17.90	
**60**	1326	84.70	15.78	
**70**	1324	83.83	14.67	
**80**	1325	83.34	13.83	
**90**	1328	82.99	13.35	

The CRE was determined from Figure 5.

### 3.5 Couch rotation method

**FIGURE 6:**
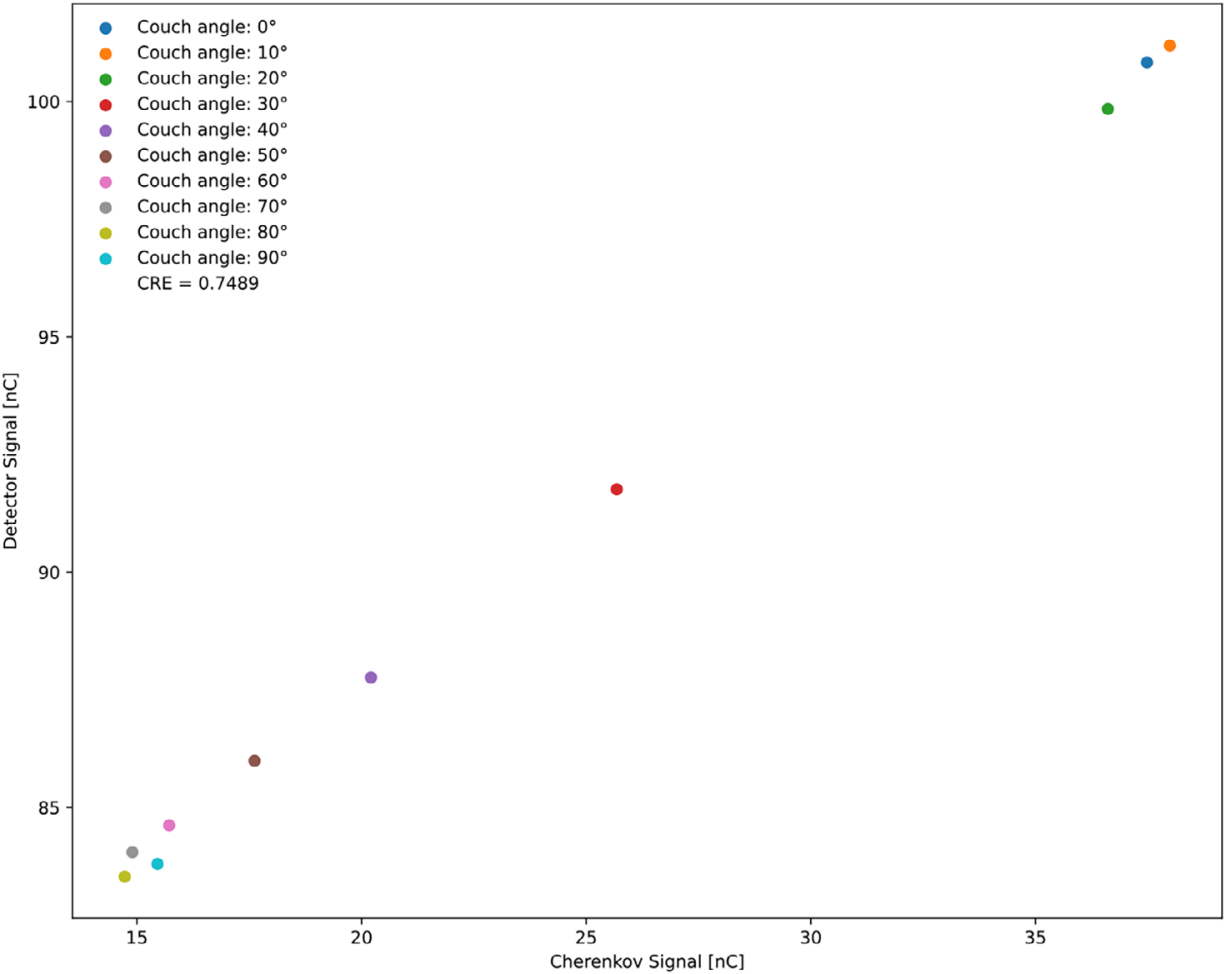
Relationship between the Cherenkov signal (x‐axis) and detector signal (y‐axis) in the couch rotation method performed on the Varian TrueBeam. The slope of this linear plot represents the CRE.

Table 5 presents the couch rotation method values, including the number of pulses, detector charge, and Cherenkov charge. The CRE, which was 0.7489, was determined from Figure 6, where the x‐axis represents the Cherenkov charges and the y‐axis represents the detector charges. By examining the slope of that plot, the CRE value was obtained.

**TABLE 5:** Measurement values from the couch‐rotation method, showing the couch angle, number of pulses, detector charge, and Cherenkov charge.

Couch angle	Number of pulses	Detector charge (nC)	Cherenkov charge (nC)	CRE
**0**	1327	100.83	37.48	0.7489
**10**	1326	101.19	37.99	
**20**	1328	99.84	36.61	
**30**	1328	91.76	25.68	
**40**	1328	87.76	20.21	
**50**	1328	85.99	17.62	
**60**	1328	84.62	15.72	
**70**	1328	84.05	14.90	
**80**	1325	83.53	14.73	
**90**	1326	83.80	15.46	

The CRE was determined from Figure 6.

## 4 DISCUSSION

CR occurs in PSDs when electrons in the detector material travel faster than the local speed of light. Although CR can be used for certain radiation measurements, it acts as noise when the PSD is coupled to an optical fiber. As a result, CR must be removed to obtain accurate readings of the scintillation signal. The 0.35 T MR‐Linac system has a cylindrical, donut‐shaped geometry containing both an MR scanner for gating and a linear accelerator, which limits the feasibility of certain CR‐removal techniques because neither the collimator nor the couch can be rotated.

**FIGURE 7:**
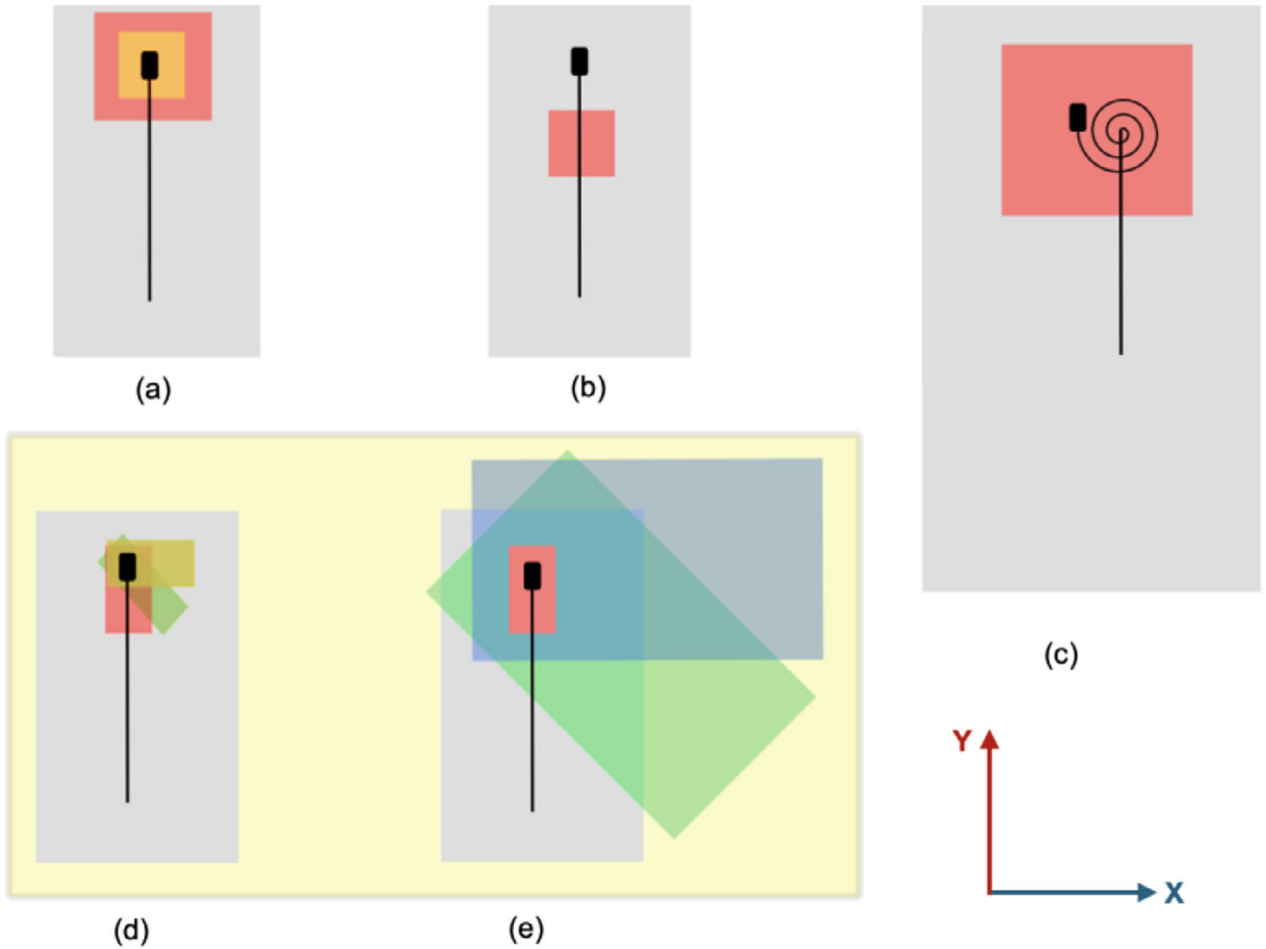
Schematic diagram of the five methods used to measure and calculate the CRE. (a) Cross‐calibration method using two field sizes, (b) Fiber‐alone method using a single field size, (c) Multiloop method with multiple coils of the fiber inside a single field, (d) Collimator‐rotation method using one rectangular field size for collimator angles from 0° to 90° in 10° increments, and (e) Couch‐rotation method using one rectangular field size for couch angles from 0° to 90° in 10° increments. Methods (a), (b), and (c) were performed exclusively on the 0.35 T MR‐Linac, while methods (d) and (e) were performed only on the Varian TrueBeam.

In this study, we evaluated five CR‐removal methods: cross calibration, fiber alone, multiloop, collimator rotation, and couch rotation. Figure 7 schematically illustrates these methods, and Table 6 summarizes their CRE values. The first three methods were tested on the 0.35 T MR‐Linac, while the remaining two (collimator and couch rotations) were examined on a Varian TrueBeam for comparison with the multiloop method.

**TABLE 6:** The CRE values for each method used in this study.

System	Method	CRE value
0.35T MR‐Linac	Cross calibration	0.7318
	Fiber alone	0.7569
	Multiloop	0.7288
Varian TrueBeam	Collimator rotation	0.7255
	Couch rotation	0.7489

Previous research on CR removal from the BP‐PSD in MR‐Linac environments has been reported by Ferrer et al.,^28^ who used multiple field sizes with assigned MU values to keep dose delivery constant. By plotting Cherenkov charge against the detector charge, they derived a linear trend and calculated CRE from its slope. Another study by Oolbekkink et al.,^30^ compared four methods for CR removal and explored approaches such as square‐field, flip‐field, rectangular‐field, and perpendicular‐field setups, adapted for MR‐Linacs where collimator rotation is not possible. However, they also evaluated collimator rotation angles of 0° and 90° on a conventional Linac for comparison, noting that using multiple rectangular fields can alter Linac output and add variability.

Our study focused on methods not requiring collimator rotation: cross calibration, fiber alone, and the multiloop method, each with advantages and drawbacks (see Table 7). Cross calibration requires precise knowledge of the Linac FOF, making it sensitive to machine output stability. In the fiber‐alone method, although the plastic detector is positioned outside the field (leaving only the fiber irradiated), scatter still delivers some dose to the scintillator, causing an elevated apparent CRE.

**TABLE 7:** Advantages and drawbacks of the five methods used in this study.

Method	Potential pros and/or cons
Cross calibration	Requires precise knowledge of output factors and is sensitive to machine output variations during measurements.
Fiber alone	Involves a small but notable dose in the scintillator due to scatter, even when placed outside the field, which can lead to a higher CRE.
Multiloop	Considered the most practical method because it does not require exact output factors, is less affected by machine variations, and uses simple analysis (no equations needed). It is also relatively error‐resistant.
Collimator rotation	Produces a CRE value similar to the multiloop method but requires rotating the collimator, which is not feasible on MR‐Linac systems. Precise rotation around the isocenter is necessary for reliable results.
Couch rotation	Some measurements deviate from a linear trend, reducing reliability. Accurate rotation around the isocenter is also required, and this method is not applicable to MR‐Linac systems.

To address these limitations, we introduced the multiloop approach, in which the fiber is looped within the field for multiple repeated measurements using the same MU. The Cherenkov (chCR) and scintillation (chPSD) signals are plotted, and the slope of their linear relationship provides the CRE (Figure 4). This method is more practical in MR‐Linac systems because it does not require rotating the couch or collimator, nor does it depend on the FOF. Additionally, looping the fiber minimizes the influence of magnetic field direction on detector orientation. Even when the Cherenkov signal exceeds the scintillation signal due to multiple loops, the linearity remains intact, simplifying slope determination and validating the technique's robustness.

We also tested collimator rotation and couch rotation on a Varian TrueBeam for comparison (Figures 5 and 6). Collimator rotation and couch rotation produced a linear relationship very similar to the multiloop method, indicating that both are viable for CRE determination. However, the multiloop method is more practical because it does not involve physically moving Linac components. Couch rotation showed greater variability and sometimes did not yield a straight line, suggesting it is less effective. Overall, the multiloop technique demonstrated high efficiency for CR removal on the 0.35 T MR‐Linac, and it can be extended to other Linac modalities.

## 5 CONCLUSION

PSDs are highly effective in MR‐Linac systems due to their small size and water‐equivalent composition, eliminating the need for correction factors. They are also largely unaffected by magnetic fields, making them advantageous for dosimetry in MR‐Linacs. However, CR manifests as noise in PSD measurements and requires removal. Given the limited design constraints of a 0.35 T MR‐Linac system, this study evaluated three CR‐removal methods for the BP‐PSD. Among these, the multiloop method demonstrated superior efficiency compared to the cross‐calibration and fiber‐alone methods. Increasing the number of loops caused the CR signal to surpass the scintillation signal while preserving linearity, and consequently, the multiloop method's CRE value approached that obtained via collimator rotation. These findings indicate that the multiloop technique is a robust, practical approach for CR removal in PSD‐based MR‐Linac applications.

## AUTHOR CONTRIBUTIONS

Mateb Al Khalifa: Conceptualization, acquisition, interpretation of data, methodology, validation, drafting the work, revising the draft, final approval of the version. Tianjun Ma: Conceptualization, interpretation of data, methodology, validation, revising the draft, final approval of the version. Haya Aljuaid: Conceptualization, interpretation of data, methodology, validation, revising the draft, final approval of the version. Siyong Kim: Conceptualization, interpretation of data, methodology, validation, revising the draft, final approval of the version. William Y. Song: Conceptualization, interpretation of data, methodology, validation, revising the draft, final approval of the version.

## CONFLICT OF INTEREST STATEMENT

The authors declare no conflicts of interest.
